# Cross-Validating the Electrophysiological Markers of Early Face Categorization

**DOI:** 10.1523/ENEURO.0317-24.2024

**Published:** 2025-01-24

**Authors:** Fazilet Zeynep Yildirim-Keles, Lisa Stacchi, Roberto Caldara

**Affiliations:** Eye and Brain Mapping Laboratory (iBMLab), Department of Psychology, University of Fribourg, Fribourg 1700, Switzerland

**Keywords:** electroencephalogram, face categorization, N170, oddball fast periodic visual stimulation, steady-state visual evoked potentials, transient event-related potentials

## Abstract

Human face categorization has been extensively studied using event-related potentials (ERPs), positing the N170 ERP component as a robust neural marker of face categorization. Recently, the fast periodic visual stimulation (FPVS) approach relying on steady-state visual evoked potentials (SSVEPs) has also been used to investigate face categorization. FPVS studies consistently report strong bilateral SSVEP face categorization responses over the occipitotemporal cortex, with a right hemispheric dominance, closely mirroring the N170 scalp topography. However, it remains unclear whether SSVEP responses can be considered a proxy for the N170 or are driven by different components. To address this question, we recorded electrophysiological signals from observers viewing face and object images during FPVS and ERP paradigms. We quantified the FPVS response in the frequency domain and extracted ERP components, including the P1, N170, and P2, from both the FPVS time domain and ERP paradigms. Our results revealed little relationship between any single ERP component and the FPVS frequency response. Only the peak-to-peak differences between N170 and P2 components consistently explained the FPVS frequency response. Our data show that the FPVS frequency response reflects a later complex neural integration rather than any isolated ERP component, such as the N170. These findings raise important methodological and theoretical considerations regarding the relationship between SSVEPs and transient ERPs. While both markers are indicative of human face categorization, they appear to capture different stages of this cognitive process.

## Significance Statement

Our study untangles the very nature of the electrophysiological neural responses of face categorization. We recorded and directly compared steady-state visual evoked potentials (SSVEPs) with transient event-related potentials (ERPs) evoked by faces and objects in human observers. Contrary to the assumption associating SSVEPs with the early N170 ERP component, we found that the N170-P2 difference was consistently associated with the SSVEPs. This finding suggests that SSVEPs in fast periodic visual stimulation (FPVS) may reflect later stages of neural processing. Our findings invite to caution when interpreting SSVEP responses, avoiding premature assumptions about their relationship with ERPs. This work highlights the need for integrated research approaches to better understand the complex interplay between SSVEPs and ERPs across different cognitive domains.

## Introduction

The human brain's ability to categorize visual objects, particularly human faces, is a fundamental question in cognitive neuroscience. Efficient face processing involves both distinguishing faces from nonface objects and generalizing across different face exemplars despite variations in size, gender, ethnicity, expression, and age.

Electroencephalography (EEG) is a popular technique for studying face categorization due to its noninvasive nature and high temporal resolution. Specifically, event-related potentials (ERPs) elicited by transient stimuli have been instrumental in understanding how intrinsic and extrinsic factors impact face categorization. The N170 ERP component, a negative deflection occurring between 130 and 200 ms on the bilateral occipitotemporal electrodes, is a widely investigated marker of early face categorization (Bötzel et al., 1995; Bentin et al., 1996; George et al., 1996). Despite their utility, ERPs have limitations such as low signal-to-noise ratio and the need for post hoc comparisons between conditions of interest.

An emerging alternative is the oddball fast periodic visual stimulation (FPVS) paradigm (Rossion, 2014; Rossion et al., 2015; Retter and Rossion, 2016), which elicits steady-state visual evoked potentials (SSVEPs; Adrian and Matthews, 1934; Regan, 1966). This method involves the presentation of a series of base stimuli at a constant frequency, with periodically intervening oddball stimuli differing along specific dimensions, like identity or expression. To investigate face categorization, nonface objects are used as base stimuli, while oddball stimuli consist of faces. This method generates distinct SSVEPs at both the base and oddball frequencies, allowing direct investigation of face processing without post hoc contrasts. Additionally, the neural responses obtained through this paradigm have high signal-to-noise ratio and their quantification does not rely on subjective choice in data analysis.

Despite its increasingly widespread usage, the exact nature of the SSVEP responses in FPVS paradigm is not fully understood. The assumption that an oddball response is functionally linked to the visual system's differential response to faces versus nonface objects is conceptually compelling. However, this raises questions about how to interpret the strength of FPVS frequency responses and whether individual differences in such responses are tied to face-specific neural processing, such as the well-established N170 ERP component. Within the context of neural facial identity discrimination, where both base and oddball stimuli are faces, the oddball response was suggested to be related to the well-known N170 ERP component (Rossion et al., 2012), as both exhibit similar scalp topographies (Rossion et al., 2012; Liu-Shuang et al., 2014) and responses to experimental manipulations like face inversion (Rossion et al., 2012). However, direct comparisons between ERP N170 and SSVEP frequency responses in the context of face categorization are lacking.

In this study, we aim to empirically and directly relate SSVEP responses to transient ERP responses to bridge this theoretical and methodological gap. Specifically, we sought to determine whether individual variations in the FPVS response amplitude during face categorization were reflected in corresponding variations in N170 amplitude across subjects. Crucially, our aim was not to pinpoint the source of the FPVS response or to claim that a single ERP component, such as the N170, could fully account for this relationship. Instead, we sought to explore which of the ERP components, including P1 and P2, account for the variance of the FPVS frequency response amplitude. Our hypothesis was that while multiple peaks in the EEG waveform likely contribute to the FPVS frequency response, the relationship with the N170 would be the strongest, suggesting an overlap in the processes these measures capture. A strong association would provide evidence that the FPVS frequency response and the N170 index similar neural mechanisms, potentially allowing one to act as a proxy for the other.

We collected EEG data from human observers viewing images of objects and faces using the oddball FPVS paradigm. Base stimuli were nonface objects presented at a high frequency, with oddball face stimuli introduced periodically. SSVEP face categorization responses were quantified by the amplitude of the oddball frequency response (i.e., the FPVS frequency response). Additionally, we extracted ERPs from the time domain of the signal corresponding to face stimuli onset.

To relate FPVS frequency responses to transient ERPs, we examined ERPs elicited by isolated faces, as well as differential face responses obtained by subtracting ERPs in response to object stimuli from those elicited by face stimuli. We also introduced a modified ERP paradigm where face stimuli were preceded by nonface images (i.e., contextual face responses), creating a more comparable context to the FPVS paradigm. Our analysis focused on the P1, N170, and P2 ERP components, as these are prominent peaks in the FPVS time-domain response and likely major contributors to the frequency domain response (Jacques et al., 2016). We also investigated peak-to-peak differences between P1 and N170, and N170 and P2, reasoning that the frequency domain response is influenced by multiple peaks and troughs. In addition to determine whether the FPVS frequency responses could be predicted by ERP components, we also assessed topographical similarities between the two types of responses.

Lastly, we explored whether these neural responses would relate to behavioral performance at a test of face recognition, the CFMT+ (Russell et al., 2009). Our findings reveal that the FPVS frequency response is not contingent on any single ERP component, such as the N170, but rather reflects a complex integration of neural processes. This study enhances our understanding of electrophysiological face categorization responses and demonstrates the practical implications of SSVEP responses in various contexts, from healthy populations to clinical assessments.

## Materials and Methods

### Overview

We recorded the electrophysiological signals of human observers who viewed natural images of objects and faces in ERP and FPVS paradigms. Then, from the same observers we recorded behavioral responses to a facial identity recognition task. We assessed how the FPVS frequency response relates to its own time-domain components, as well as components extracted from three ERP measures including isolated faces in which faces were presented in isolation, differential faces in which isolated nonface object images were subtracted from the isolated face images, and contextual faces in which faces were preceded by four nonface object images. Moreover, we investigated how the time components extracted from the FPVS paradigm relate to those extracted from the ERP paradigms. Lastly, we also assessed the relationship between the electrophysiological signals of ERP and FPVS paradigms and the behavioral performance.

### Participants

Thirty-four participants were tested (four males, one left-handed; mean age, 22.6 ± 2.9; range, 19–30). All participants reported normal or corrected-to-normal vision, and none had reported to have a history of psychiatric or neurological disorders. Observers participated in an EEG session, composed of two ERP and one FPVS experiments. Twenty-nine participants also took part in a behavioral task following the EEG session. Prior to participating in the study, all participants gave their written consent. The study was approved by the local ethics committee and conformed to the Declaration of Helsinki.

One participant was rejected due to excessive noise in the EEG signal resulting in too few trials per condition. The results from 33 participants were retained for the final analysis (22.7 ± 2.9 years, four males, one left-handed).

### Experimental design

#### Stimuli and apparatus

Stimuli were displayed at the center of a VIEWPixx/3D monitor (1,920 × 1,080 pixel resolution, 120 Hz refresh rate) on a light gray background using the Psychtoolbox in Matlab 2017b (The MathWorks). Stimuli consisted of photographic images of various man-made objects and images of human faces. The same stimuli were used in previous studies investigating face categorization using the oddball FPVS paradigm (de Heering and Rossion, 2015; Rossion et al., 2015). All objects and faces were presented without isolation from their original backgrounds. While centered within the display, they varied in size, viewpoint, lighting, and background. The stimuli were converted to grayscale, resized to 320 × 320 pixels, and adjusted for pixel luminance and contrast using the SHINE toolbox (Willenbockel et al., 2010). Notably, given that this normalization process is applied to the entire image, the facial features in these natural images retained intentional variations in local luminance, contrast, and power spectrum. The stimuli subtended ∼6.6 by 6.6 degrees of visual angle.

EEG was acquired by means of BioSemi ActiView software with a BioSemi ActiveTwo amplifier system and 128 Ag-AgCl Active electrodes. Offset was lowered and maintained below 30 mV relative to the common mode sense (CMS) and driven right leg (DRL) by slightly abrading the scalp and adding saline gel. The signal was digitalized at a sampling rate of 1,024 Hz. Digital triggers were sent by means of a VPixx Technologies screen.

#### Procedure

**Figure 1.**
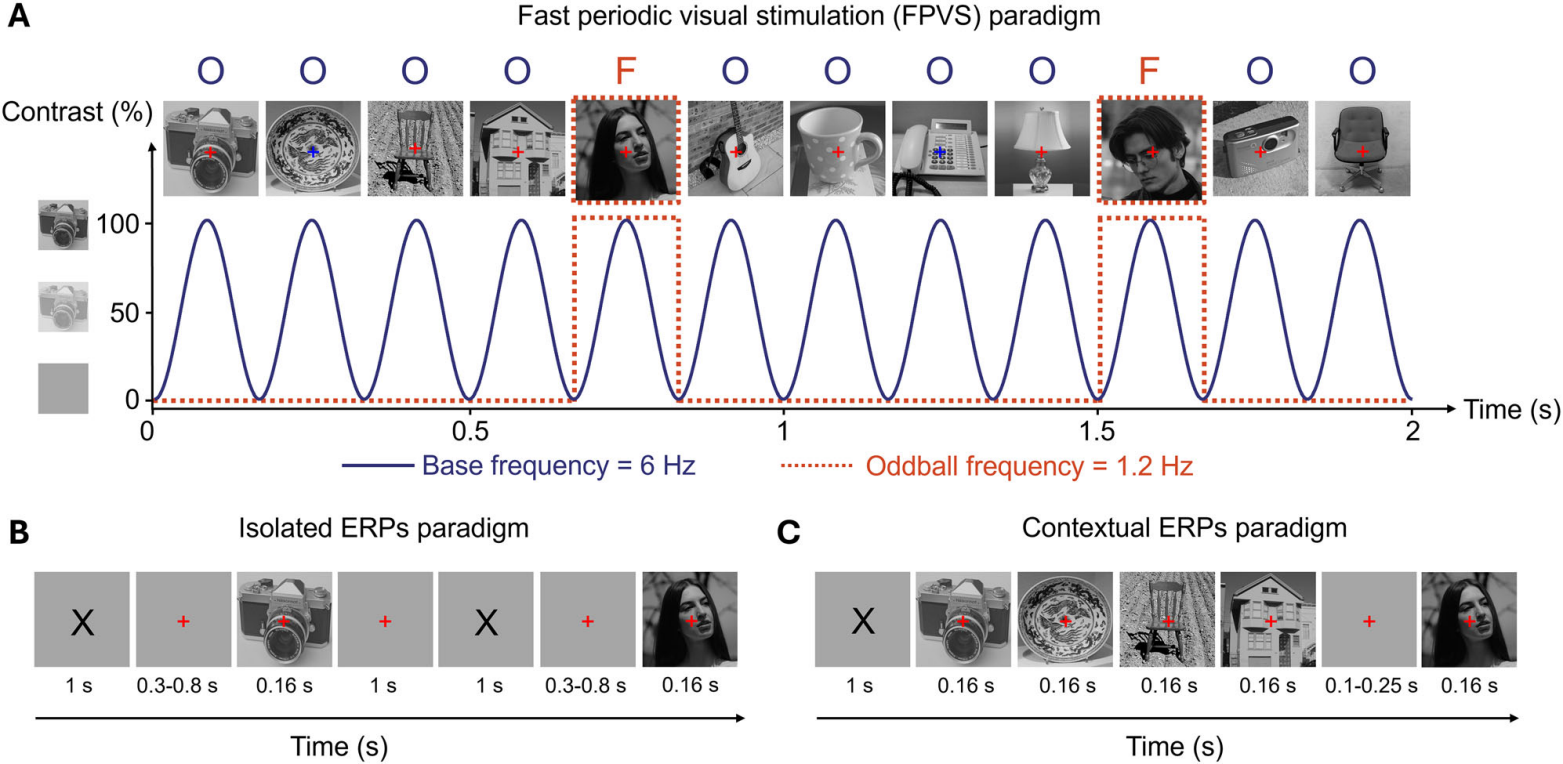
A schematic illustration of the FPVS and ERP paradigms. ***A***, Natural images of objects were presented at 6 Hz following a sinusoidal contrast modulation. Face images were inserted every five stimuli, corresponding to a frequency of 1.2 Hz (=6 Hz/5). ***B***, Natural images of objects and faces were presented in isolation. ***C***, A face image was preceded by four object images. Note that face images in the figure differ from those used in our experiment due to copyright restrictions.

A schematic illustration of the FPVS (Fig. 1*A*) and ERP (Fig. 1*B*,*C*) paradigms as employed is presented in Figure 1. After the implementation of the EEG cap, participants were seated comfortably at a distance of 75 cm from the computer screen. Participants’ head positions were stabilized with a head and chin rest to maintain viewing position and distance constant. Participants were instructed to refrain from any movements during the experiment. Due to the sensitive nature of the ERP signals, participants first completed the ERP experiments and then performed in the FPVS experiment. The order of the isolated and contextual ERP experiments was randomized for each participant. In all experiments, participants were instructed to fixate on a red cross located on the center of the screen while continuously monitoring the stimuli presented. In all experiments, participants’ task was to detect brief (500 ms) color changes (red to blue) of this fixation cross. In the FPVS paradigm, color changes occurred 10 times within every trial. In the isolated ERP paradigm, 24 trials with color changes occurred randomly among 160 trials with no color changes. In the contextual ERP paradigm, 12 trials with color changes occurred randomly among 80 trials with no color changes. This task was orthogonal to the manipulation of interest in the study and was used to ensure that the participants maintained a constant level of attention throughout the experiment.

In the FPVS paradigm, within each trial, a sequence of visual stimuli was presented through sinusoidal contrast modulation at a base frequency of 6 Hz; hence, each stimulus lasted 0.166 s (i.e., 1,000 ms/6). This base stimulation frequency rate was selected because it elicits large periodic brain responses to faces in adults (Alonso-Prieto et al., 2013; Retter et al., 2021). Each trial lasted 68 s, in which the stimuli were presented in sequences lasting 64 s, which were flanked by a 2 s fade-in at the beginning of the sequence and by a 2 s fade-out at its end. The 2 s buffer time at the beginning and end of each stimulation sequence was used to avoid abrupt onset and offset of the stimuli, which could elicit eye movements. This resulted in a total of 409 images per trial, all of which were different between trials. In each sequence, four nonface objects were presented consecutively, followed by a face, with all stimuli randomly chosen from their respective categories. This arrangement resulted in the oddball faces being presented at a frequency of 6/5 = 1.2 Hz. Consequently, the EEG amplitude specifically at this oddball face stimulation frequency and its harmonics (2.4 Hz, 3.6 Hz, etc.) served as a measure of the brain's ability to discriminate between faces and objects, as well as its ability to generalize across different facial stimuli. Trials with activity exceeding 100 µV of absolute amplitude were repeated. Participants completed four trials in the FPVS paradigm. During recording, the experimenter visually monitored the signal and repeated the trials for which large deflections were observed. During this task, subjects were allowed to blink whenever it was necessary.

In the isolated ERP paradigm, each trial began with a black X mark (≈1° of visual angle) presented at the center of the screen for 1 s, which was then replaced by a red fixation cross (≈0.3° of visual angle) presented for an interval of random duration between 0.3 and 0.8 s. A face or nonface object was then presented for 0.166 s. The offset of the face or nonface stimulus was followed by an intertrial interval of 1 s. Participants completed 80 trials with face stimuli and 80 trials with object stimuli.

The contextual ERP paradigm followed the same procedures as the isolated ERP paradigm except for the following differences. Instead of a single visual stimulus, in each trial a face stimulus, presented for 0.166 s, was preceded by four nonface object images, each presented for 0.166 s. A variable interval duration of 0.1–0.25 s was inserted in between the presentation of face and nonface objects to avoid expectation-related modulation of the neural signal. Participants completed 80 trials.

During both transient ERP paradigms, participants were instructed to refrain from blinking during the presentation of the visual stimuli. During EEG recording the experimenter visually monitored the signal and the participant and repeated trials clearly contaminated by eyeblinks.

A longer version of the Cambridge Face Memory Test (CFMT; Duchaine and Nakayama, 2006) with an added section of 30 difficult trials containing heavily degraded images (CFMT+; Russell et al., 2009) was employed at the end of the EEG session to provide a behavioral measure of facial identity recognition. The test features grayscale cropped male face stimuli with six target and 46 distractor identities. Participants initially are familiarized with images of target identities from diverse viewpoints and subsequently are asked to identify the target image in a three-alternative forced-choice task. As the test progresses, trials become progressively more difficult due to manipulations in illumination, orientation, visual noise, and information availability. We recorded the number of correct responses of each participant across the 102 trials.

### FPVS preprocessing and analysis

#### Preprocessing

All EEG analyses were conducted using Letswave 5 (Mouraux and Iannetti, 2008) and Matlab 2017b (The MathWorks). Continuous EEG data were first bandpass filtered to exclude frequencies below 0.1 Hz and above 100 Hz using a fourth-order Butterworth filter. The signal was then downsampled to 512 Hz. Then, for each observer, 2 × 66 s epochs, which included 2 extra seconds pre- and poststimulation, were extracted. An independent component analysis using a square mixing matrix algorithm was conducted to remove blink-related noise from each participant's data (up to two components were selected based on their topography and time-course). Data were then visually inspected and electrodes that presented systematic noise-related deflection over multiple trials were interpolated (with no more than 5% of electrodes interpolated per participant). Subsequently, the data were re-referenced to a common average reference and cropped to an integer number of oddball's cycles starting 2 s after stimulation onset and ending 2 s before stimulation offset (=30,720 bins).

#### Frequency domain analysis

**Figure 2.**
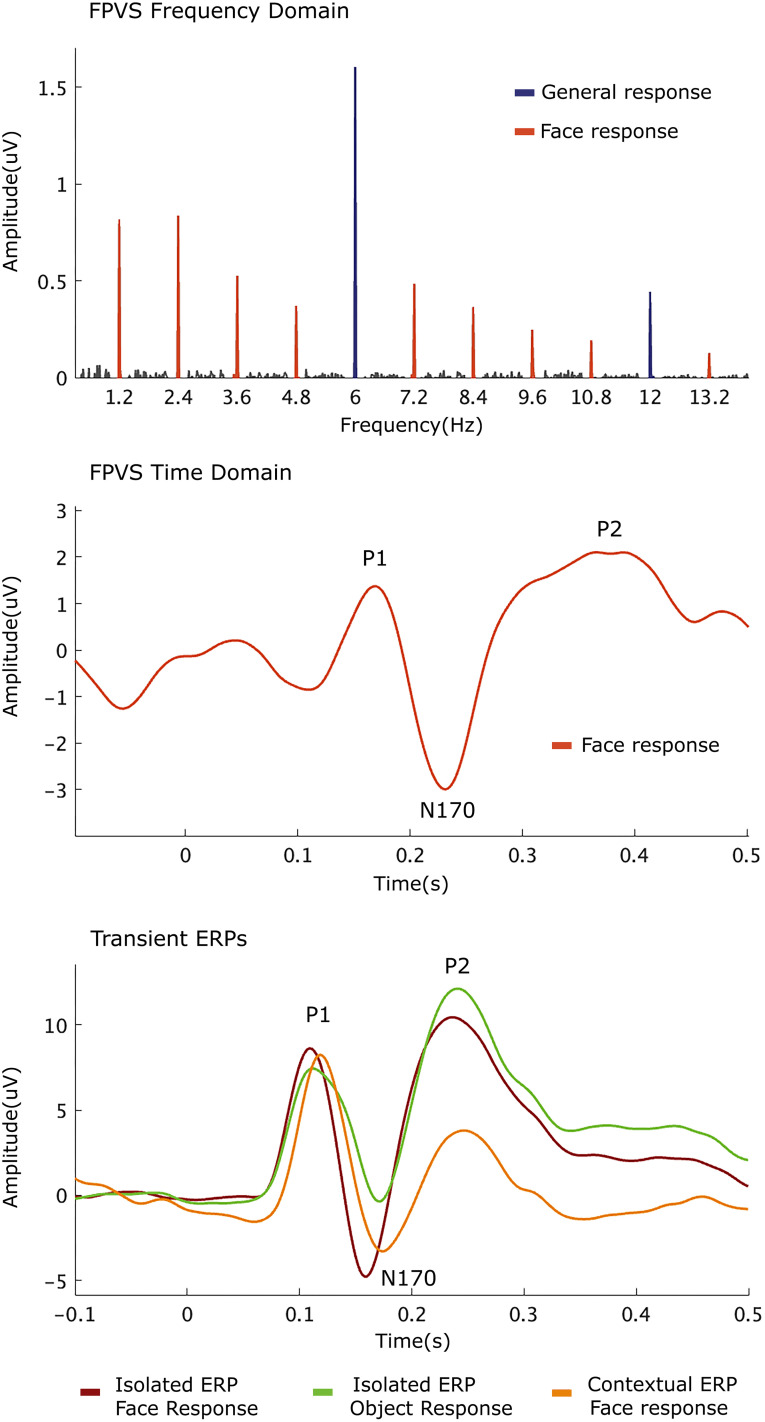
Baseline-corrected amplitudes averaged for signals at five right occipitotemporal channels showing significant responses at the oddball stimulation frequency (1.2 Hz) and its harmonics (up to 13.2 Hz), as well as at the base stimulation frequency (6 Hz) and its harmonics (significant up to 24 Hz but shown until 12 Hz in the figure; top panel). ERP waveforms averaged for signals at five right occipitotemporal channels showing peaks at P1, N170, and P2 for FPVS time domain (middle panel) and transient ERPs (bottom panel).

**Figure 3.**
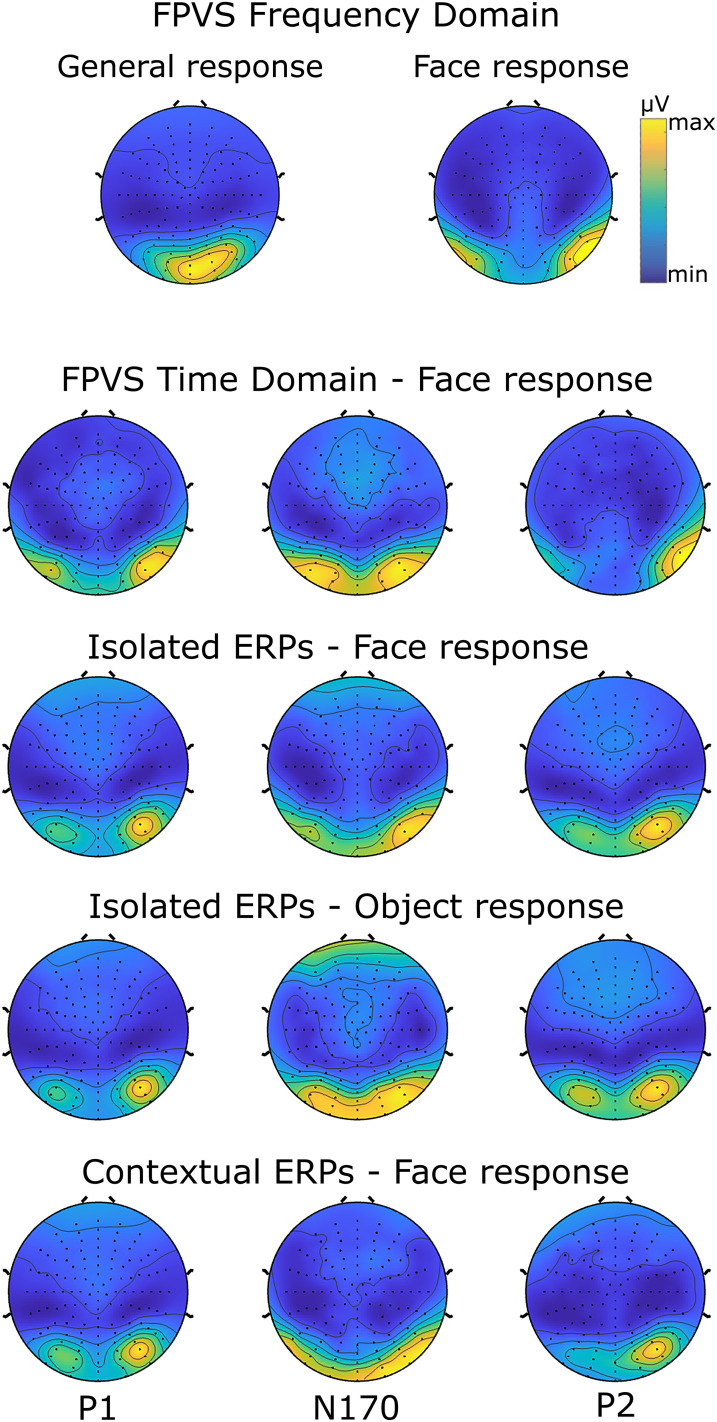
Grand averaged topographical maps for FPVS frequency domain responses (i.e., general and face-selective), and P1, N170, and P2 peaks in FPVS time domain and transient ERP responses. Absolute values of each peak are taken to facilitate visual comparison between peaks in topographical similarity analysis. Topographical maps for general and face-selective responses in FPVS frequency domain is based on the sum of corrected amplitudes at significant harmonics. For the topographical similarity analysis results between the topography of the FPVS frequency responses and the topographies at P1, N170, and P2 latencies, see Extended Data Figure 3-1.

The periodic oddball responses were analyzed in the frequency domain. Following re-referencing, the amplitude of EEG responses in the frequency domain was computed using Matlab's built-in fast Fourier transform (FFT) function (N/2 points with normalized amplitudes). To account for baseline variations, baseline correction was applied to all of the resulting amplitude spectra by subtracting the average of the surrounding 20 bins from each frequency bin, excluding the two immediately neighboring bins. Signal averaging was performed across 20 (A9–A16, A22–A29, B6–B9) and 10 occipitotemporal electrodes (A10–A12, B7–B11, D31–D32) to encompass channels sensitive to general (base) and face categorization (oddball) responses, respectively. A10, A15, A23, A28, B7, B10, B11, D31, and D32 in BioSemi 128-channel system approximately corresponds to PO7, O1, Oz, O2, PO8, P10, P8, P7, and P9 in the 10–20 system, respectively (channel coordinates can be found at https://www.biosemi.com/headcap.htm). Considering that the periodic response to stimulation is spread over multiple harmonics (i.e., integer multiples of the stimulation frequency), the relevant range of frequency harmonics was determined independently for base and oddball frequencies. *Z*-scores were calculated after averaging across subjects and electrodes to identify significant harmonics, with significance determined by *z*-scores exceeding 2.32 (*p* < 0.01, one-tailed) for two consecutive harmonics (*Z*-scores were computed following the same logic as the baseline-correction). Significant responses at the oddball frequency (1.2 Hz) and its harmonics were indicative of face categorization, while responses at the base frequency (6 Hz) reflected a combination of face-related processing and general visual responses. Based on this threshold the oddball response, which indexes implicit neural face categorization, was quantified by summing the first 11 oddball harmonics (i.e., 1.2–13.2 Hz), with the exclusion of the 5th and 10th harmonics due to their overlap with the base stimulation frequency rate (Fig. 2, top panel). The base response remained significant until the fourth harmonic (i.e., 6–24 Hz). As our primary focus was on the face categorization response rather than the overall visual response, we solely assessed the fundamental base frequency (i.e., 6 Hz) to ensure the validity of our experimental manipulation regarding fixated visual input. Grand averaged topographical maps for oddball and base frequency responses are shown in Figure 3 (top panel).

10.1523/ENEURO.0317-24.2024.f3-1 Figure 3-1 The strength of the relationship between the topography of the FPVS frequency responses and the topographies at P1, N170, and P2 latencies, extracted both from the time domain of the FPVS response as well as from the isolated and contextual ERP paradigms. Download Figure 3-1, DOCX file.

#### Time-domain analysis

The periodic oddball responses were also analyzed in the time domain. The re-referenced EEG data was low-pass filtered with a cutoff frequency of 30 Hz using a fourth-order Butterworth filter. A notch filter (with a width of 0.05 Hz) was then applied to selectively eliminate the influence of the base stimulation frequency and its first five harmonics (ranging from 6 to 30 Hz) from the time-domain waveforms. Subsequently, the filtered data were segmented into smaller epochs lasting 1.67 s each, with the initial epochs during the trial's fade-in period discarded. Baseline correction was performed on these smaller epochs by subtracting the mean response amplitude observed during the 100 ms preceding the presentation of the face image. For each trial, 70 epochs were acquired per participant. These epochs were averaged for each participant and then grand averaged for display of time-domain data (Fig. 2, middle panel).

From these averaged waveforms, ERP components were extracted for each participant at five electrode sites in the right hemisphere, chosen for their known sensitivity to face-selective effects (B7–B11). Consistency in electrode site selection across conditions and individual ERP components was crucial for meaningful regression model comparisons (see below, Statistical analysis). Visual inspection revealed substantial topographical overlap both among the FPVS time-domain components (Fig. 3) and between the FPVS time-domain components and the ERP components, aligning with prior literature highlighting face-related responses primarily over occipitotemporal sites, with a dominance in the right hemisphere (Rossion, 2014; Lochy et al., 2019). EEG waveforms revealed three components: a positive component peaking at ∼160 ms (“P1”), followed by a large negative component peaking at a 220 ms latency (“N170”), and finally by a large positive component that peaks at ∼410 ms (“P2”). Despite the delay in components’ latencies (compared with transient ERP components), we will keep referring to these components as P1, N170, and P2 for the sake of simplicity and consistency throughout our manuscript. Finally, the peak-to-peak differences between N170 and preceding P1 (P1-N170) and between N170 and succeeding P2 (N170-P2) were calculated to capture the integrative effects of these components.

### ERP preprocessing and analysis

#### Preprocessing

Continuous EEG data were bandpass filtered between 0.5 and 30 Hz (Butterworth filter, order 4) and downsampled to 512 Hz. Trials with color changes and button presses were excluded from the analyses. The downsampled data was then segmented into epochs centered on stimulus onset (−2 to 2 s). Electrode coordinates and spline files were assigned for building the 2D scalp maps and the 3D head plots, respectively. Epochs containing blink artifacts within the time window from −0.1 to 0.5 s were removed. Subsequently, EEG data were segmented into 0.6 s epochs (−0.1 to 0.5 s relative to stimulus onset) for each condition (isolated faces, isolated objects, and contextual faces). Baseline correction was performed by subtracting the amplitude in the 0.1 s prestimulus period. Noisy channels with systematic deflections exceeding 100 μV over multiple trials were reconstructed using linear interpolation from neighboring clean channels, with no more than 5% of channels interpolated per participant. The interpolation of occipital channels was maintained consistently across all ERP and FPVS EEG data analysis to prevent biasing of the signal of interest. Epochs containing amplitude exceeding ±75 μV within −0.1 to 0.5 s time window were discarded. A common average reference computation was applied to all channels. Finally, averaging was performed across 66 epochs for all subjects, with the subject having the fewest epochs in a specific condition serving as the threshold. Group averaged data of isolated face, isolated object, and contextual ERP waveforms are shown for display of time-domain data (Fig. 2, bottom panel).

#### Time-domain analysis

After preprocessing, the EEG waveforms were utilized to extract ERP components for each participant and condition separately (i.e., face and object stimuli conditions in the isolated ERP, face stimulus in the contextual ERP). Differential ERPs were obtained by subtracting the isolated ERP components to objects from isolated ERP components to face stimuli. ERPs were extracted within specific time windows corresponding to P1, N170, and P2 at five electrode sites in the right hemisphere (B7–B11), as in the FPVS time domain. Visual inspection of topographies indicated considerable overlap across conditions and individual ERP components (Fig. 3). Peak amplitudes for P1, N170, and P2 were observed approximately between 80 and 140 ms, 150 and 230 ms, and 180 and 250 ms, respectively. These peak amplitudes were then determined and extracted individually for each condition, participant, and electrode of interest. Additionally, as in the FPVS time domain, the peak-to-peak differences between N170 and preceding P1 (P1-N170) and between N170 and succeeding P2 (N170-P2) were calculated for all conditions. Topographical maps for each peak and ERP condition, excluding the differential ERPs (see below, Statistical analysis), were created as in the FPVS time domain.

### Statistical analysis

Statistical analyses were performed in R statistical software (version 3.6.3). Generalized linear models (GLMs) with Gamma family and log link function were employed to assess the associations between the amplitude of FPVS oddball frequency responses and the response amplitude from FPVS time data, isolated ERP, differential ERP, and contextual ERP. Specifically, the FPVS oddball frequency responses were regressed against the P1, N170, P2, P1-N170, and N170-P2 responses separately for each of the four data types (FPVS time data, isolated ERP, differential ERP, and contextual ERP). This approach yielded a total of 20 models (five predictors by four data types). The models were built using the average amplitude across the five right occipitotemporal electrodes.

Bayes factors were used to assess the strength of evidence for the associations between FPVS oddball frequency responses and the responses from FPVS time, isolated ERP, differential ERP, and contextual ERP analyses. Bayes factors of each model were computed by comparing the predictor model with the intercept-only model (i.e., model with no fixed effect) using the model.comparison() function from the flexplot package (Fife et al., 2021). This comparison helps determine whether including predictors improves model fit and supports any hypothesized association. A Bayes factor greater than 1 suggests evidence in favor of the alternative hypothesis (i.e., the model with a predictor) over the null hypothesis (i.e., the intercept-only model), while a Bayes factor less than 1 indicates evidence in favor of the null hypothesis. Given the necessity to account for multiple comparisons and to mitigate the increased risk of false positives, an evidence boundary of Bayes factor greater than or equal to 10 was chosen to denote significance of the predictor model compared with the intercept-only model (Schönbrodt and Wagenmakers, 2018; Stefan et al., 2019). This higher evidence boundary indicates strong evidence for the presence of an effect, thus reducing the likelihood of erroneously identifying associations. Models passing this significance threshold (BF_10 _≥ 10) were further contrasted with each other to reveal the hierarchical relationship between them in explaining the FPVS frequency response. The adjusted deviance-based *R*-squared values for each model were computed using the adj*R*^2^() function from the glmtoolbox package (Vanegas et al., 2023) to assess how effectively the models fit the data. Importantly, the adjusted deviance-based *R*-squared value can range from negative infinity to 1. A value of 1 indicates a perfect fit, while 0 suggests no improvement over using the response variable. Negative values occur when the model fits worse than no predictors.

To further understand whether the responses elicited in the FPVS paradigm and transient ERP paradigms are equivalent, we also explored the relationship between the ERPs extracted from the time domain of the FPVS data and transient ERPs. The main goal was to compare the responses triggered by these two types of stimulations in the same domain, namely, the time domain. To this aim, we used linear models (LMs) to regress the FPVS responses in the time domain on transient ERPs. This was done for each peak and peak-to-peak separately resulting in 15 models (five predictors by three data type). Bayes factors were computed and the significance threshold (i.e., BF_10 _≥ 10) was applied as previously. The *R*-squared values were computed using the model.comparison() function.

We assessed the stability of the ERP measures by examining how well they predicted each other. The primary aim was to evaluate the consistency of these ERPs before comparing them to the distinct responses observed in the FPVS paradigm. To this aim, we employed LM to regress one type of ERP on another. This was done for each peak and peak-to-peak separately resulting in 25 models (five predictors by five data type, including isolated object ERPs). Bayes factors and *R*-squared values were computed and the significance threshold (i.e., BF_10 _≥ 10) was applied as previously.

To assess the overall topographical similarities across measures, we considered 44 occipitotemporal electrodes. The peak amplitudes of P1, N170, and P2 components were extracted for contextual and isolated ERP paradigms as well as from the time-domain response of the FPVS paradigm. For each peak, we first identified, within measure, the strongest electrode of the 44 occipitotemporal electrodes. Using the latency of its maximum absolute amplitude, we then defined a window of 26 ms surrounding the peak (∼13 ms before and after the peak). Finally, for each electrode, we computed the mean peak within this time window. Importantly, within the context of ERPs, peaks can be either positive or negative due to the presence of dipoles. However, the relevant aspect of a peak is the absolute amplitude. Hence, to allow the comparison with FPVS frequency responses, we converted the ERP responses into their absolute values. Differential ERPs, which involve subtracting object responses from face responses, were excluded from the topographical similarity analysis. This is because taking absolute values of these differences can obscure the true signal, making it difficult to interpret meaningful topographical patterns compared with direct ERPs. To assess the strength of the topographical relationship, we used generalized linear mixed models (GLMERs) with Gamma family and log link to regress the FPVS frequency response onto that of any of the time-domain responses. We included a random-effect intercept based on subject identity, to capture the individual variability in topographical similarity, allowing for heterogeneity between individuals. Then, we determined whether the relationship between two topographies was significant by contrasting an intercept-only model, with a model including the predictor term. Pseudo *R*^2^ was computed using the trigamma function (Nakagawa et al., 2017). Bayes factors were computed and the significance threshold (i.e., BF_10 _≥ 10) was applied as previously.

Finally, we explored the relationship between observers’ behavioral performance on the CFMT+ and their EEG responses across all paradigms. GLMs (for the FPVS frequency response) and LMs (for the time-domain components) were employed to regress EEG responses onto the accuracy scores of CFMT+. Bayes factors and *R*-squared values were computed and the significance threshold (i.e., BF_10 _≥ 10) was applied as previously.

## Results

### FPVS frequency response versus FPVS time domain and transient ERP responses

**Figure 4.**
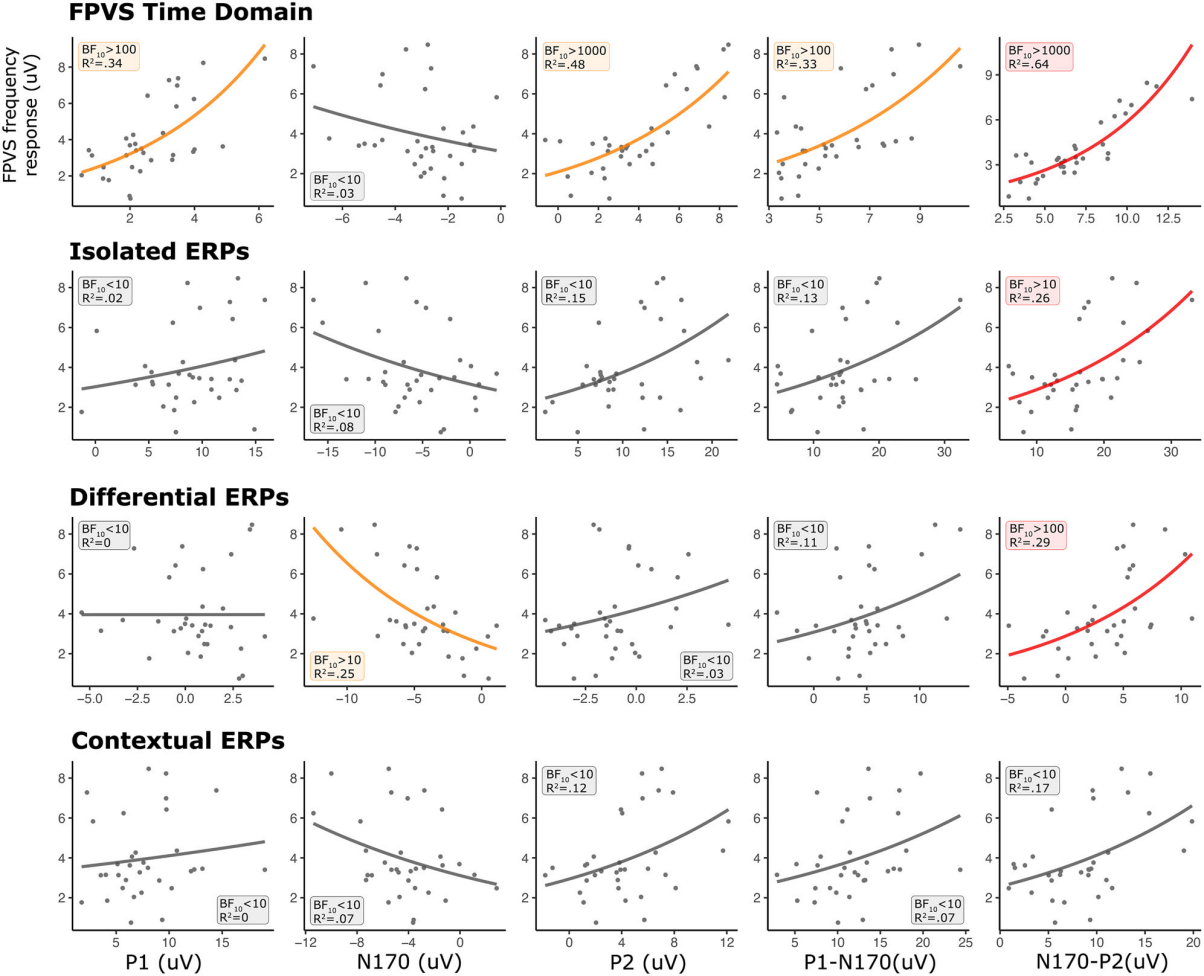
The relationship between the FPVS frequency response and time-domain components (P1, N170, P2, P1-N170, and N170-P2) in FPVS time, isolated ERPs, differential ERPs, and contextual ERPs. The individual dots denote individual subjects. The colored lines (orange and red) denote significance of the predictor model against the intercept-only model. Red lines denote the model with the highest *R*^2^ value within a specific paradigm.

The models depicting the relationship between the amplitude of FPVS oddball frequency responses and time-domain components across paradigms are shown in Figure 4. Significant associations were observed between FPVS oddball frequency responses and specific time-domain components in each paradigm (except contextual ERPs).

Within the FPVS time components, all components were significantly associated with FPVS frequency response (BF_10 _> 100, *R*^2 ^≥ 0.33), with the exception of the N170 component (BF_10 _< 10). Within the isolated ERP components, the only significant association was found between the FPVS frequency response and N170-P2 difference (BF_10 _= 54.0, *R*^2 ^= 0.26). Within the differential ERP components, a significant association was found for the N170 component (BF_10 _> 10, *R*^2 ^= 0.25) as well as for the N170-P2 difference (BF_10 _> 100, *R*^2 ^= 0.29). Lastly, within the contextual ERP components, no significant association was found, with largest association observed for N170-P2 difference (BF_10 _= 6.94, *R*^2 ^= 0.17).

The hierarchical analysis of models is conducted to provide insights into their respective associations with the FPVS frequency response. The N170-P2 in FPVS time demonstrated the strongest association, followed by the P2 in FPVS time, then the N170-P2 in isolated and differential ERP, N170 in differential ERP, and the P1 and the P1-N170 in FPVS time, all of which showed comparable association strength.

Given that the peak-to-peak differences result from subtracting the peak amplitudes of the individual components, we explored potential connections between the significant peak-to-peak differences and their constituent components. Our regression analysis revealed a high correlation between the N170-P2 in FPVS time and the P2 component (BF_10 _> 1,000, *R*^2 ^= 0.654), but not with the N170 component (BF_10 _= 5.875, *R*^2 ^= 0.192). The N170-P2 in isolated ERP (P2: BF_10 _> 1,000, *R*^2 ^= 0.654; N170: BF_10 _> 1,000, *R*^2 ^= 0.654), the N170-P2 in differential ERP (P2: BF_10 _> 100, *R*^2 ^= 0.352; N170: BF_10 _> 1,000, *R*^2 ^= 0.697), and the P1-N170 in FPVS time (P1: BF_10 _> 100, *R*^2 ^= 0.322; N170: BF_10 _> 1,000, *R*^2 ^= 0.575) were strongly correlated with each of their constitute components.

### FPVS time domain versus transient ERP responses

**Figure 5.**
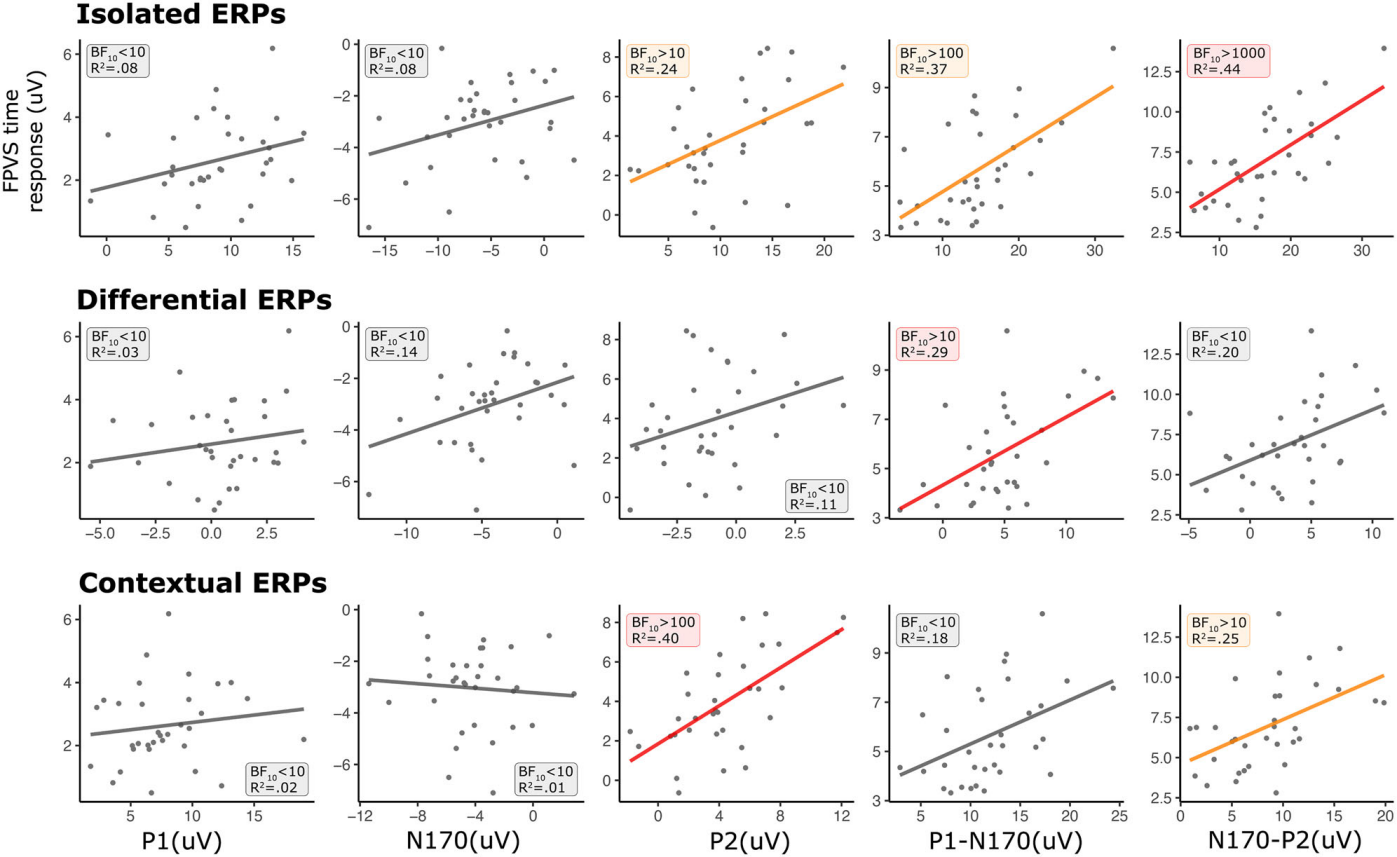
The relationship between the FPVS time-domain components and transient ERP components. The individual dots denote individual subjects. The colored lines (orange and red) denote significance of the model against the intercept-only model. Red lines denote the model with the highest *R*^2^ value within a specific ERP condition. For the models depicting the relationship between transient ERP measures, see Extended Data Figure 5-1.

The models depicting the relationship between the components extracted from the time domain of the FPVS data and those obtained from transient ERPs are shown in Figure 5. Within the isolated ERP condition, significant associations were found for the P2 (BF_10 _> 10), P1-N170 (BF_10 _> 100), and N170-P2 (BF_10 _> 1,000). Within the differential ERP condition, a significant association was observed for the P1-N170 difference (BF_10 _> 10). Finally, within the contextual ERP condition, a significant relationship was found for the P2 component (BF_10 _> 100) and for the N170-P2 different (BF_10 _> 10). In line with results above, this pattern underscores the significance of the N170-P2 difference, which emerges as a consistent predictor across paradigms.

10.1523/ENEURO.0317-24.2024.f5-1 Figure 5-1 The models depicting the relationship between transient ERP measures. The first condition name in each title corresponds to x axis while the second name corresponds to y axis. The individual dots denote individual subjects. The coloured lines (orange and red) denote significance of the model against the intercept-only model. Red lines denote the model with the highest R^2^ value within a specific ERP correlation. Strong correlations are observed between the isolated face, contextual face, and isolated object ERP measures, indicating that ERP components are stable and consistent across different experimental conditions. Download Figure 5-1, TIF file.

### Isolated versus contextual ERP responses

The models depicting the relationships between the different ERP measures—isolated face ERPs, contextual face ERPs, isolated object ERPs, and differential ERPs—are shown in Extended Data Figure 5-1. Significant associations (BF_10 _> 10) were found across the isolated face, contextual face, and isolated object ERP conditions, indicating strong correlations between these measures. The differential ERPs did not correlate well with the other ERP measures (BF_10 _= 0.27–7.62, *R*^2 ^= 0.026–0.2).

### Topographical similarity analysis

Topographical similarity analysis showed that all topographies at P1, N170, and P2 latencies extracted from the time domain of the FPVS response as well as from the isolated and contextual ERP paradigms are significantly related of the topography of the FPVS frequency response (all BF_10 _> 100; Extended Data Fig. 3-1).

### Neural face categorization responses versus behavioral performance

Finally, the regression models assessing the relationship between the accuracy scores in the CFMT+ revealed that the accuracy in the CFMT+ was not significantly associated with any of the EEG responses including the frequency response and time-domain responses.

## Discussion

This study aimed to determine if the face categorization response elicited by the FPVS paradigm is related to the face-sensitive N170 ERP component or other neural responses. We explored the relationship between the FPVS frequency domain response and ERP components from FPVS in the time domain, isolated faces, differential face responses, and contextual face responses. We focused on the P1, N170, and P2 peaks, as well as the P1-N170 and N170-P2 differences. Given the right hemisphere's dominance in face processing, we concentrated on a right occipitotemporal region of interest (ROI; for a review, see, Rossion and Lochy, 2022). Our findings suggest that the N170 ERP component has minimal explanatory power over the FPVS frequency response, which is most strongly and consistently associated with the N170-P2 peak-to-peak difference across paradigms.

### FPVS frequency domain versus FPVS time domain

We found a significant relationship between ERP components from the FPVS time domain and the frequency response for all components except the N170, with the N170-P2 difference showing the greatest explanatory power. Analysis performed to assess the relationship between the N170-P2 difference and its individual constituents showed that the peak-to-peak difference significantly relates to the P2 but not the N170 component. This further suggests that the relationship between the FPVS frequency response and the N170 obtained from its time domain is limited and the two measures might relate to different processes. The stronger correlation of the FPVS frequency response with the N170-P2 difference might be due to the fast Fourier transform (FFT) method, which assesses sinusoidal waves’ presence. The amplitude of peak-to-peak ERP difference likely impacts frequency amplitude more than a single ERP peak. However, why the P1 and P2 peaks, but not the N170, are significantly related to the frequency response needs further explanation.

One explanation could be that the neural response to face stimuli in FPVS varied among face exemplars and observers. We presented six stimuli per second using natural images, embedding faces in diverse backgrounds, with variations in head orientation, expression, age, or gender. This variability, along with attention lapses, blinking, and fatigue, might have made some face stimuli harder to detect. As shown by Latinus and Taylor (2006), difficulties in face processing can modulate ERP components’ latency (see also Jemel et al., 2003; Németh et al., 2014). Their findings indicate that detecting a face in a Mooney-face, which poses a greater challenge for detection due to their simplified nature and lack of detailed features compared with photographic faces, caused a greater delay in the N170 compared with the P1 and P2 components (Latinus and Taylor, 2006). Greater latency variability leads to less periodic occurrence of a component, making it less conspicuous in the frequency domain. Moreover, averaging responses following each face presentation can result in less precise peaks if latency variations are indeed present.

A recent study by Retter et al. (2020) found that when observers fail to detect a face among nonface objects, the FPVS time-domain response lacks discernible ERP components. However, there is no information on difficult-to-detect faces, which might be indicated by longer latencies or higher uncertainty. Future research should investigate the impact of stimulus-driven difficulties on the FPVS responses in both time and frequency domains. Slower presentation frequencies or segregated images of faces and nonface objects could reduce neural face categorization difficulties. If this hypothesis was confirmed, it could suggest that the frequency response can reflect different face categorization stages across different observers. Concretely, this could mean that the FPVS frequency response of some observers is influenced by the N170 component to a greater extent than the response of other individuals, which in turn would make the comparison across individuals questionable.

Finally, when considering the relationship between the FPVS frequency response and the components extracted from the time domain, it is important to keep in mind that the frequency response is a measure that results from the summation of multiple responses at both the fundamental frequency and its significant harmonics. While we did not address this aspect in the current study, we believe that future research should investigate how different harmonics might relate to the time-domain components. This line of investigation could shed further light on the nature of the FPVS response and its relationship with well-known ERP components.

### FPVS frequency domain versus transient ERP responses

The comparison of the FPVS frequency response to transient ERPs revealed that the N170-P2 difference had the highest explanatory power for both differential and isolated face responses. Along with the results from FPVS time domain, this suggests that the frequency response integrates early and later face-related responses.

No ERP component from the contextual face response showed a significant relationship with the FPVS frequency response. We used a short and variable interstimulus interval (ISI) between the last object and the face stimulus to reduce face onset predictability. This short ISI might have caused object response contamination and inconsistent component onset across the face responses, introducing noise that overpowered the signal. This could also explain the weaker amplitude of both the N170 and P2 components in the contextual condition compared with the isolated condition. It is important to note that the lack of correlation between the FPVS frequency response and the contextual face response does not indicate a limitation of the FPVS paradigm. The FPVS paradigm optimally captures automatic face processing and it is robust against stimulus confounds, as any confounding effects would need to appear periodically to impact the oddball response. In contrast, our contextual condition, where participants might anticipate a face after every four pictures, could relate to controlled face processing. Thus, the differences observed between these paradigms might denote the distinct nature of each measure. Our results should be interpreted within this context rather than as a limitation of one approach over the other.

In terms of the N170 component, we found a significant relationship only for the one extracted from the differential face response, likely because this ERP response aligns closely with the FPVS frequency response: a differential response free from nonface object contamination.

Importantly, the correlations with differential ERPs should be interpreted with caution. As in previous studies (Eimer, 1998, 2000; Goffaux et al., 2003; Flevaris et al., 2008), we calculated differential ERPs to provide a face-sensitive neural response not contaminated by object-related activities. However, this approach relies on the strong assumption that object-related populations respond to the same extent to both objects and faces. Only under this hypothesis it is then justified to expect that subtracting the response to object from the one to faces would leave a face-specific response. In fact, while this subtraction approach is commonly and effectively used in fMRI across voxels to distinguish face- versus nonface responses (Kanwisher et al., 1997; Rossion et al., 2003; Caldara et al., 2006; Grill-Spector et al., 2006; Caldara and Seghier, 2009), its suitability in the context of scalp EEG studies remains highly debatable. Specifically, EEG recordings are characterized not only by their temporal dynamics but, more critically, by low spatial resolution. The electrophysiological signals captured by the electrodes reflect the summed activity of multiple neural populations, which introduces ambiguity in the spatial localization of the recorded signals. Consequently, it is arguable whether this differential response can fully capture face-specific neural activity. At the very least, any differential ERPs should be interpreted with great caution.

### FPVS time domain versus transient ERP responses

To attempt to better understand the relationship between the FPVS frequency response and transient ERP components, we regressed the ERP components extracted from the different ERP measures on those obtained from the FPVS time-domain data. We found no relationship between the P1 and the N170 obtained from the time domain of the FPVS response with any of the transient ones. However, the P1-N170 difference extracted from the FPVS time domain were related to the one recorded in the isolated and differential ERP paradigms. Finally, the P2 as well as the N170-P2 difference extracted from the FPVS time domain were significantly related to those recorded in the isolated and contextual ERP paradigms. The lack of a relationship for the two early components, P1 and N170, might be related to the important methodological differences between the FPVS paradigm and traditional ERPs. Specifically, it is likely that the response to faces during FPVS is contaminated by residual activity in response to the preceding object. The FPVS time domain-transient ERP relationship might still emerge for the later P2 component as the contaminating activity might weaken at those latencies. These results suggest that while the P1 and N170 extracted from the FPVS time domain might appear at the same latencies as traditional ones and share similar topographies, they might indeed reflect slightly different processes. Further research is needed to validate this hypothesis, for example, by exploring the same relationship at different stimulation frequencies, which would lead to different degrees of contamination on the face response.

### Isolated versus contextual ERP responses

The strong correlations observed between the isolated face, contextual face, and isolated object ERP measures suggest that these ERP components are stable and consistent across different ERP paradigms. This stability provides a solid foundation for interpreting the FPVS results, as it indicates that the ERPs are reliable measures of neural responses.

We found that the differential ERPs did not correlate well with the other ERP measures. This is expected as isolated face and object ERPs were highly correlated. We calculated the differential ERPs by subtracting the isolated object ERPs from the isolated face ERPs. This subtraction then potentially led to removal of the correlated elements between faces and objects, leaving the differential ERPs largely independent of the isolated face ERPs and resulting in the observed lack of correlation.

### Topographical similarities

Our topographical analysis revealed that all three ERP peaks—P1, N170, and P2—across different paradigms were significantly related to the topography of the FPVS frequency response. This is likely due to the fact that these responses differentiate from one another only in subtle aspects. Importantly, Hauk et al. (2021) investigated the brain sources associated with face-selective FPVS frequency responses and their corresponding time-domain evoked components (P1, N170, and P2). They found that peak activity was consistently located in the posterior brain regions of both hemispheres, with a more widespread distribution in the right hemisphere. While a similar posterior pattern was observed in the time-domain components, there were variations in the spread and spatial location of activity: N170 was more anterior than P1, and P2 activity shifted back toward the occipital pole (Hauk et al., 2021). Our results are consistent with these findings as we found strong evidence of a topographical relationship between the FPVS frequency response and all ERP peaks. This alignment suggests that the topographical similarities we observed may be rooted in the shared underlying neural mechanisms that Hauk et al. (2021) identified, where the spatial dynamics of these ERP components contribute to the face-selective FPVS frequency response.

The topographical similarity analyses were restricted to comparing the topography of FPVS frequency responses with the topographies of isolated and contextual ERP peaks, rather than differential ERP peaks. This decision was based on how differential ERPs are derived—specifically, by subtracting object responses from face responses. For the analysis, we considered a large posterior region consisting of 44 electrodes, which inevitably capture face responses to varying degrees. As a result, after the subtraction process, electrodes that originally showed stronger responses for objects than faces would display negative values.

In the case of isolated and contextual ERPs, positive and negative peaks arise from dipole orientations, with the critical information being their magnitude, or distance from zero. To address this, we took the absolute value of each electrode's response and related it to the FPVS responses, which reflect periodic activity at the face presentation rate. However, applying this approach to differential ERPs would complicate distinguishing face-specific from object-specific responses and make it more challenging to interpret their relationship with FPVS frequency responses.

### Neural face categorization responses versus behavioral performance

We investigated the relationship between the neural face categorization responses and behavioral performance. Retter et al. (2020) found that conditions with higher face detection performance had stronger FPVS frequency responses. However, predicting individual behavioral performance from neural responses remains unclear. We correlated performance in a face recognition task with FPVS frequency response and various ERP components but found no significant relationships. However, our study was limited to one behavioral task indexing facial identity recognition. Other tasks addressing face categorization more closely might yield stronger correlations with these neural responses. More importantly, future studies should investigate how different neural markers of face processing such as the N170 ERP component and the FPVS frequency response can predict behavioral performance in face processing tasks. This assessment would provide tools to better understand the neural processes captured by these neural measures.

### The implications for the relationship between SSVEPs and ERPs

Our findings offer key insights into the relationship between steady-state evoked potentials (SSEPs) and transient ERPs. Traditionally, SSEPs and ERPs in high-level cognition have been studied separately, leading to a fragmented understanding of their interaction.

Previous research primarily examined SSEPs and ERPs in low-level processes in auditory (Saupe et al., 2009; Zhang et al., 2013) and visual domains (Müller and Hillyard, 2000; Heinrich and Bach, 2003). Müller and Hillyard (2000) found correlations between SSVEP amplitudes and N1/N2 ERP components with flickering LEDs, while Heinrich and Bach (2003) found no such correlation with random-dot motion stimuli. Zhang et al. (2013) noted a correlation between different SSEP amplitudes but not with transient ERPs.

However, the relationship between SSEPs and ERPs in high-level cognitive processes remains largely unexplored. Our study challenges the assumption that SSEPs straightforwardly reflect underlying ERP components in complex tasks like face categorization, revealing a stronger correlation between the FPVS frequency response and the N170-P2 peak-to-peak difference than with single ERP components such as the N170. This finding suggests that the assumption that the FPVS response merely represents individual ERP components may be oversimplified, particularly for high-level cognitive tasks.

This caution extends to other cognitive processes like word recognition (Lochy et al., 2024), semantic categorization (Stothart et al., 2017), and working memory (Nurdal et al., 2021). Our results highlight the need for nuanced interpretations of SSEPs, recognizing their unique contributions and avoiding premature assumptions about their relationship with ERPs. Overall, this study underscores the importance of integrated research approaches to understand the interplay between SSEPs and ERPs across cognitive domains.

### Conclusion

We investigated the neural mechanisms indexed by the FPVS frequency response in neural face categorization. Our results challenge the assumption that the FPVS frequency response directly reflects the N170 component, suggesting instead that it represents a later stage in face processing. Generalizability of these results across stimulus manipulation and experimental conditions should be investigated in future studies, which should further clarify the relationship between FPVS frequency response and well-established ERP components.

Our findings emphasize the N170-P2 difference as the primary predictor of the FPVS frequency response, contrasting with the limited correlation with the N170 component. This suggests that the FPVS technique captures complex neural responses, necessitating thorough investigation to fully understand its mechanistic and theoretical implications. Future studies should explore how task constraints (e.g., face discrimination) might modulate the relationship between FPVS frequency and face-sensitive N170 responses.

In conclusion, our study highlights the need for cautious interpretation of FPVS frequency responses and their relationship to traditional ERP components, advocating for more integrated research approaches to fully understand neural face categorization.

## Data Availability

The preprocessed EEG data and behavioral data that support the findings of this study are available on OSF (https://osf.io/fdkhg/?view_only=e185d69e904d48cfa90b2bb14c3cf9cb).

## Synthesis

Reviewing Editor: Ifat Levy, Yale School of Medicine Decisions are customarily a result of the Reviewing Editor and the peer reviewers coming together and discussing their recommendations until a consensus is reached. When revisions are invited, a fact-based synthesis statement explaining their decision and outlining what is needed to prepare a revision will be listed below. The following reviewer(s) agreed to reveal their identity: George Stothart. Note: If this manuscript was transferred from JNeurosci and a decision was made to accept the manuscript without peer review, a brief statement to this effect will instead be what is listed below.

Two reviews have been received. They both recommend revision of the manuscript. The detailed comments of both reviewers are attached. In summary, two major points need careful consideration:

1. The FPVS responses are an average over time and are not necessarily expected to reflect one single ERP component.

2. There should be more emphasis on comparing topographies (or even sources) than peak amplitudes.

When revising the manuscript, please attach a point-by-point response to each of the reviewer's comments.

Reviewer 1:

This EEG study investigates the relationship between event-related potentials (ERPs) and fast periodic visual stimulation (FPVS) responses in human face categorization. It is still not established whether FPVS responses reflect the N170 or other specific ERP components. By recording electrophysiological signals from observers viewing face and object images, the authors found small correlations between the amplitudes of any single ERP component and the corresponding FPVS frequency response. The most important predictor turned out to be the peak-to-peak difference between N170 and P2 components. Thus, it appears that the FPVS frequency response reflects a later and more complex response.

This is a clear and well-written study using standard ERP and FPVS methodology. I would still like to ask for some clarifications before I can fully make up my mind.

1. The relationship between ERP and FPVS responses is indeed not yet clear and an interesting topic for research. However, it is not clear to me how important this is for the relevance of FPVS responses, or for their interpretation as "early" or "late." It is clear that the FPVS oddball and base responses collapse signals across time into one peak in the frequency spectrum. Thus, we gain SNR at the expense of temporal resolution. The response will necessarily be a mix of ERP "components" (if this term is really meaningful) and anything in between, plus possibly extra FPVS-specific responses. The current analyses do not analyze this in much detail, and only use correlations of amplitudes across participants. While this provides some evidence, it is not enough to conclude whether or how the FPVS responses reflect ERP components. This would require a more detailed analysis of the topographies, and ideally source distributions. I appreciate that source estimation may be out of the scope of this study. But the authors could correlate the topographies with each other. They could even build a linear model consisting of topographies of multiple ERP components and test how well different components fit the FPVS response (just note that you would have to take absolute values in order to account for power in the frequency domain). From Figure 3, the FPVS frequency and time domain as well ERP topographies look very compatible to me.

2. Following up from the previous point, it may not be surprising that the P2-N170 is the best predictor since it reflects a combination of components, as does the FPVS response itself. This is somewhat part of the argument around line 419. The fact that it includes the N170 component means that it is likely to contribute to the FPVS response. Thus, I think this study's conclusions are too pessimistic. I don't know what "minimal explanatory power" (line 412) means. The fact that it doesn't show the largest correlation doesn't mean it is not important. In my view, the results suggest quite strongly that there is significant overlap between FPVS responses in the frequency and time domain as well as ERPs.

3. Please note that source estimation of face-selective FPVS responses and the corresponding ERPs has already been reported in https://psycnet.apa.org/record/2021-83696-001, which should be discussed.

4. I find the "contextual" face response problematic. Participants will expect a face after every four pictures. This will draw attention to the face stimuli, while in the FPVS runs face processing will be more "automatic", which some authors see as advantage of the FPVS paradigm. It has also been argued that the FPVS paradigm is more robust against stimulus confounds, since they would have to appear periodically (thus be present in almost every stimulus) in order to affect the oddball response. Thus, some differences between this and more conventional paradigm may actually reflect its advantages.

Minor points:

• line 181: How were "trials with excessive muscle noise" identified?

• page 10: Are there 10-20-label equivalents of electrode names "A...", which I assume are Biosemi-specific? Was signal averaging performed across both hemispheres for base and oddball frequency?

• page 10: Why was a threshold of p<0.01 chosen rather than 0.05?

Reviewer 2:

The authors present a useful methodological paper that attempts to unpick the time series contributors to FPVS oddball responses. The paper is thorough and the research well conducted and presented. My comments are listed below, in order of importance, sorry for the use of CAPS but it was the only way to emphasise given the text editor available when submitted my review!:

1. The oddball, or f+, response in FPVS studies is typically characterised by averaging across multiple harmonics of the oddball stimulation frequency, and in this study this is also the case. Given that f+ is a composite of 11 harmonics, it seems highly unlikely that it would EVER correlate that well with a single ERP peak. It is by definition a composite response, and to my mind that is one of its strengths. It is POSSIBLE that a single ERP peak generated the f+ response but in that case I'd expect to see very few significant harmonics. I don't see this as a barrier to this paper being published, but I think the point should be emphasised in the discussion as it is integral to the design of the study.

2. It is very difficult to draw many meaningful conclusions from the comparison of the FPVS time series with the traditional time series. The overlapping stimuli epochs in the FPVS time series compromises the accurate measurement of ERPs so significantly you're not making a fair comparison. I do think time series analyses can be useful in FPVS studies, i.e. the data isn't useless, it's just not a fair comparison to compare them to standard ERPs.

3. To benchmark the FPVS regressions, it would be interesting to see how well the isolated, differential and contextual ERPs predicted each other. This would give a sense of how stable the measures are, before them comparing them to a very different response in f+. If they predict each other very well, then great, you have a nice stable ERP and showing that FPVS doesn't predict them is informative. If however they don't predict each other well then it would suggest you have an ERP with a lot of intra-individual variability depending on design.

4. Linked to my first point, have you considered looking at the predictive power of individual harmonics to the N170? There would be a multiple comparisons issue to control for, but given that f+ is a composite response it would be interesting to see if there were individual harmonics that tied with specific ERPs.
