# Supplementary material for: Cross-Validating the Electrophysiological Markers of Early Face Categorization
Source: eNeuro. 2025 Jan 24;12(1):ENEURO.0317-24.2024. doi: 10.1523/ENEURO.0317-24.2024 (PMC11781244; doi:10.1523/ENEURO.0317-24.2024)
Supplement: Figure 3-1 — The strength of the relationship between the topography of the FPVS frequency responses and the topographies at P1, N170, and P2 latencies, extracted both from the time domain of the FPVS response as well as from the isolated and contextual ERP paradigms. Download Figure 3-1, DOCX file. [file eneuro-12-ENEURO.0317-24.2024-s003.docx]

**Figure 3-**1. Topographical similarity analysis results.

|  |  | BF_10_ | Marginal R^2^ | Conditional R^2^ |
| --- | --- | --- | --- | --- |
| FPVS-TIME | P1 | 2.72x10^35^ | 0.30 | 0.36 |
|  | N170 | 2.53x10^34^ | 0.26 | 0.31 |
|  | P2 | 1.61x10^40^ | 0.32 | 0.33 |
| ISOLATED | P1 | 8.85x10^23^ | 0.23 | 0.29 |
|  | N170 | 3.55x10^26^ | 0.23 | 0.29 |
|  | P2 | 5.79x10^27^ | 0.26 | 0.29 |
| CONTEXTUAL | P1 | 1.15x10^21^ | 0.20 | 0.26 |
|  | N170 | 1.43x10^23^ | 0.19 | 0.24 |
|  | P2 | 1.04x10^10^ | 0.10 | 0.13 |
