# Supplementary figures and images for: Cross-Validating the Electrophysiological Markers of Early Face Categorization
Source: eNeuro. 2025 Jan 24;12(1):ENEURO.0317-24.2024. doi: 10.1523/ENEURO.0317-24.2024 (PMC11781244; doi:10.1523/ENEURO.0317-24.2024)

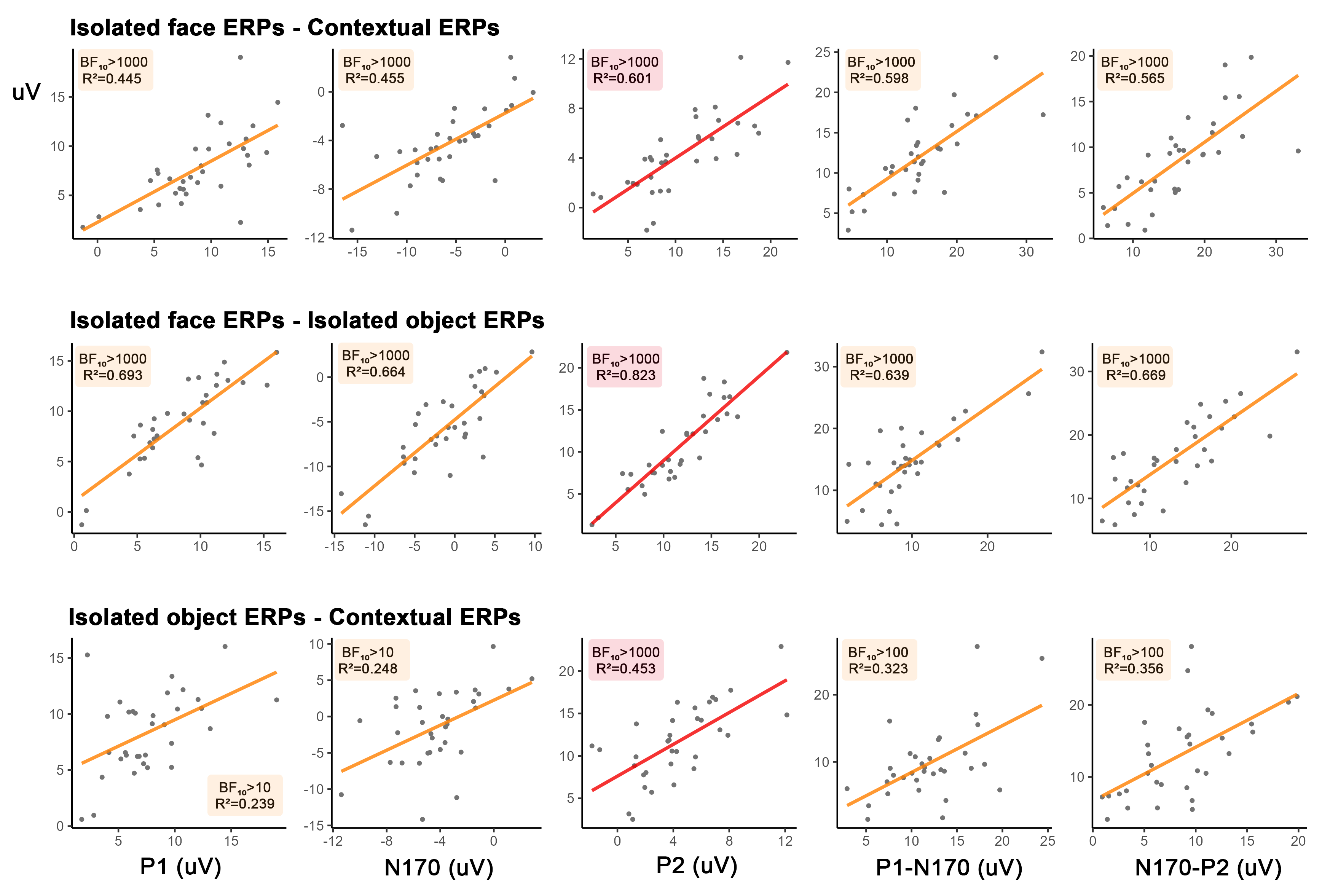

Supplement: Figure 5-1 — The models depicting the relationship between transient ERP measures. The first condition name in each title corresponds to x axis while the second name corresponds to y axis. The individual dots denote individual subjects. The coloured lines (orange and red) denote significance of the model against the intercept-only model. Red lines denote the model with the highest R2 value within a specific ERP correlation. Strong correlations are observed between the isolated face, contextual face, and isolated object ERP measures, indicating that ERP components are stable and consistent across different experimental conditions. Download Figure 5-1, TIF file. [file eneuro-12-ENEURO.0317-24.2024-s002.tif]
